# Metoclopramide or domperidone improves post-pyloric placement of spiral nasojejunal tubes in critically ill patients: a prospective, multicenter, open-label, randomized, controlled clinical trial

**DOI:** 10.1186/s13054-015-0784-1

**Published:** 2015-02-13

**Authors:** Bei Hu, Heng Ye, Cheng Sun, Yichen Zhang, Zhigang Lao, Fanghong Wu, Zhaohui Liu, Linxi Huang, Changchun Qu, Lewu Xian, Hao Wu, Yingjie Jiao, Junling Liu, Juyu Cai, Weiying Chen, Zhiqiang Nie, Zaiyi Liu, Chunbo Chen

**Affiliations:** Department of Critical Care Medicine, Guangdong General Hospital, Guangdong Academy of Medical Sciences, 106 Zhongshan Er Road, Guangzhou, 510080 Guangdong PR China; Department of Critical Care Medicine, The First Affiliated Hospital of Guangdong Pharmaceutical University, 19 Nonglinxia Road, Guangzhou, 510080 Guangdong PR China; Department of Critical Care Medicine, Jiangmen Wuyi Traditional Chinese Medicine Hospital, 30 Huayuandong Road, Jiangmen, 529000 Guangdong PR China; Department of Critical Care Medicine, Guangdong Armed Police Hospital, 106 Yanling Road, Guangzhou, 510507 Guangdong PR China; Department of Critical Care Medicine, The First Affiliated Hospital of Shantou University Medical Collage, 57 Changping Road, Shantou, 515041 Guangdong PR China; Department of Critical Care Medicine, Guangdong Yunfu People’s Hosipital, 120 Huanshidong Road, Yunfu, 527300 Guangdong PR China; Department of Critical Care Medicine, Cancer Center of Guangzhou Medical University, 78 Hengzhigang Road, Guangzhou, 510095 Guangdong PR China; Department of Cardiovascular Epidemiology, Cardiac Surgery, Guangdong Cardiovascular Institute, Guangdong General Hospital, Guangdong Academy of Medical Sciences, 106 Zhongshan Er Road, Guangzhou, 510080 Guangdong PR China; Department of Radiology, Guangdong General Hospital, Guangdong Academy of Medical Sciences, 106 Zhongshan Er Road, Guangzhou, 510080 Guangdong PR China

## Abstract

**Introduction:**

The use of prokinetic agents on post-pyloric placement of spiral nasojejunal tubes is controversial. The aim of the present study was to examine if metoclopramide or domperidone can increase the success rate of post-pyloric placement of spiral nasojejunal tubes.

**Methods:**

A multicenter, open-label, randomized, controlled trial was conducted in seven hospitals in China between April 2012 and February 2014. Patients admitted to the intensive care unit and requiring enteral nutrition for more than three days were randomly assigned to the metoclopramide, domperidone or control groups (1:1:1 ratio). The primary outcome was defined as the success rate of post-pyloric placement of spiral nasojejunal tubes, assessed 24 hours after initial placement. Secondary outcomes included success rate of post-D1, post-D2, post-D3 and proximal jejunum placement and tube migration distance. Safety of the study drugs and the tubes during the entire study period were recorded.

**Results:**

In total, 307 patients were allocated to the metoclopramide (n = 103), domperidone (n = 100) or control group (n = 104). The success rate of post-pyloric placement after 24 hours in the metoclopramide, domperidone and control groups was 55.0%, 51.5% and 27.3%, respectively (*P* = 0.0001). Logistic regression analysis identified the use of prokinetic agents, Acute Physiology and Chronic Health Evaluation (APACHE) II score <20, Sequential Organ Failure Assessment (SOFA) score <12 and without vasopressor as independent factors influencing the success rate of post-pyloric placement. No serious drug-related adverse reaction was observed.

**Conclusions:**

Prokinetic agents, such as metoclopramide or domperidone, are effective at improving the success rate of post-pyloric placement of spiral nasojejunal tubes in critically ill patients.

**Trial registration:**

Chinese Clinical Trial Registry ChiCTR-TRC-12001956. Registered 21 February 2012.

## Introduction

Nutritional support is an important part of the treatment of critical illnesses, and enteral nutrition is recommended prior in critically ill patients [1]. Studies have shown that post-pyloric feeding may reduce gastric retention, regurgitation and aspiration, resulting in decreased incidence of complications such as aspiration pneumonia [2-4]. Moreover, post-pyloric feeding can improve the efficiency of enteral nutrition and reduce the time of nutritional support [5,6]. Guidelines therefore recommend post-pyloric feeding as the preferred nutritional support route in critically ill patients with intolerance to gastric feeding. However, it is not easy to implement post-pyloric feeding conventionally [7,8].

Despite a number of different methods to place feeding tubes, no universal standard method is available [9]. Currently, fluoroscopic and endoscopic methods are mainly used in feeding tube placement [10,11]. Fluoroscopic and endoscopic methods are reliable but costly, often requiring transfer of patients and delaying early initiation of post-pyloric feeding in patients with critical illness [12]. Moreover, radiation exposure is not negligible when fluoroscopic guidance is used [13]. By-the-bedside nasojejunal tube placement is possible in more than 80% of patients [14]. No specialized equipment is required, and patient transport and delay in nutrition may be avoided [15]. Various bedside techniques, including air insufflation, pH assisted and spontaneous passage with or without motility agents, are available to facilitate transpyloric feeding tube passage [16].

Recently, clinicians have attempted to use a spiral nasojejunal tube to implement post-pyloric feeding in critical illness [17]. Spiral nasojejunal tubes consist of a polyurethane tube, 145 cm long, with a spiraled extremity and are designed to facilitate spontaneous migration into the jejunum with the assistance of gastrointestinal motility after successful gastric placement. Research has shown that spiral nasojejunal tubes are preferable to straight tubes for bedside unguided post-pyloric feeding in patients with impaired or normal gastric emptying [18]. However, the overall success rate of post-pyloric placement is significantly lower than placement by endoscopy [19].

Given that self-advancing tubes depend on gastrointestinal motility, researchers have attempted to use prokinetic agents to improve the success rate of post-pyloric placement of spiral nasojejunal tubes. Metoclopramide is a dopaminergic blocker with antiemetic and gastroprokinetic effects. It is commonly used to treat nausea and vomiting, to facilitate gastric emptying and to treat migraine-associated gastric stasis [20]. Domperidone is a specific blocker of peripheral dopamine receptors. It is used to relieve nausea and vomiting, to increase food transit through the stomach by increasing gastrointestinal peristalsis and to increase lactation [21,22]. However, the results of research into the use of prokinetic agents are controversial. An early prospective randomized trial showed no significant relationship between administration of metoclopramide and successful tube placement [23]. On the other hand, Lai and colleagues achieved a success rate of 57% using a spiral tube compared with 0% using a straight tube in patients with abnormal gastric emptying who received prior administration of 10 mg metoclopramide [18]. However, the results of these studies should be viewed with caution due to their small sample sizes, differences in baseline data of the study population and inconsistent dosage of prokinetic drugs. In addition, the use of domperidone to facilitate nasojejunal tube placement has not yet been reported.

The aim of the present multicenter randomized controlled trial was therefore to determine the efficacy of metoclopramide or domperidone in promoting post-pyloric placement of spiral nasojejunal tubes in critically ill patients.

## Methods

### Study design

A prospective, multicenter, open-label, randomized, controlled clinical trial was conducted in the ICUs of seven university hospitals. The study received approvals from ethic committees of Guangdong General Hospital (study organizer, approval number GDREC2011132H) and other participating centers (listed in Acknowledgements). Written informed consent was obtained from each patient or from the next of kin for patients unable to consent. The trial was registered with the Chinese Clinical Trial Registry (ChiCTR-TRC-12001956) [24].

### Patients

Consecutive patients admitted to ICUs between April 2012 and February 2014 and requiring enteral nutrition for more than 3 days were enrolled in the present trial. Eligibility criteria were: patients admitted to the ICU; age ≥18 years; and requiring enteral nutrition for more than 3 days. Exclusion criteria were: history of percutaneous gastrostomy or gastrojejunostomy; intubation intolerance; esophageal varices or strictures; previous major gastroesophageal surgery (for example, esophagectomy or gastrectomy); pregnant; or history of allergy to metoclopramide, domperidone, or meglumine diatrizoate.

Patients who fulfilled all eligibility criteria were eligible for randomization. Computer-generated block randomization (block size = 6) according to the sequence of recruitment was used to randomize patients. Clinicians who enrolled and treated the subjects were not involved in data collection. Eligible patients were randomly assigned in a 1:1:1 ratio at each hospital to receive metoclopramide, domperidone or nothing after telephone verification with the randomization center.

### Intervention

A self-propelled feeding tube, 145 cm long, made of radiopaque polyurethane (CH10; Flocare Bengmark, Nutricia, the Netherlands) was used in this trial. According to the method described by Berger and colleagues [17], the feeding tube was inserted in the supine position, with the head tilted at 30°. The tube was straightened using the stylet, and the stylet and tube lumen were lubricated with 10 ml paraffin. The tube was then inserted 50 to 55 cm down into the larger nostril. The position was confirmed by air injection to the stomach. The stylet was then pulled out by about 25 cm with gentle tugs until loose, and the tube was inserted down 75 to 80 cm. Before removing the stylet, the position of the tube was again confirmed. The stylet was removed while maintaining the tube at the nose level with the other hand and pulling it out. The tube was fixed on the patient’s face with a free loop of about 40 cm to allow migration, and the tube was confirmed by abdominal X-ray scan after 24 hours, before feeding.

According to the trial protocol, patients allocated to the metoclopramide group received 20 mg (or 10 mg in cases of renal insufficiency) metoclopramide intravenously 10 minutes before tube insertion. In the domperidone group, domperidone (20 mg tube feeding × 4/day) was administered by the tube immediately after it was successfully placed into the stomach. No drug was used before and after tube insertion in the control group. The treating physicians dictated the patients’ care according to their condition, including the ventilation regimen, blood glucose control, resuscitation and hemodynamic support, organ support, sedation or analgesia as needed and adequate nutrition.

### Endpoints

The primary efficacy endpoint was defined as the success rate of post-pyloric placement (post-pyloric means reaching the first portion of the duodenum or beyond) of the spiral nasojejunal tube assessed 24 hours after insertion. Secondary outcomes included the success rate of post-D1 (defined as reaching the second portion of the duodenum or beyond), post-D2 (defined as reaching the third portion of the duodenum or beyond), post-D3 (defined as reaching the fourth portion of the duodenum or beyond) and proximal jejunum placement and tube migration distance (defined as the nose scale of the tube recorded 24 hours after tube placement minus the initial nose scale immediately recorded after successful gastric placement).

The position of the tube was confirmed with air insufflation, and on abdominal X-ray scan 24 hours after tube insertion. To help confirm the tube position, additional hydrosoluble contrast injection of meglumine diatrizoate was administered via the tube before radiography, if necessary. Confirmation of tube placement using X-ray examination was routinely performed after placement, but there were no additional X-ray examinations. The placement results were examined by an expert group of ICU clinicians and radiologists who were independent and blinded to the allocation sequence. Progression was considered successful when the tube had at least reached the duodenum, and according to the part of duodenum it had at least reached. The exact location of the tube was confirmed, including stomach, first (D1), second (D2), third (D3) and fourth (D4) portions of the duodenum, and proximal jejunum.

### Safety

Adverse events of the study drugs and the tubes were recorded in all patients within 24 hours after tube insertion. Safety assessment was based on the comparison of all available information obtained from the three groups with respect to detected outliers in laboratory safety data, drug-related adverse events (assessed and recorded by the investigator) and tube insertion-related adverse events (assessed and recorded by the investigator).

### Data collection

Once patients were enrolled, data including demographic characteristics, diagnosis and concomitant medication were collected. The following clinical parameters were recorded after enrollment: severity of illness as assessed by the Acute Physiology and Chronic Health Evaluation II (APACHE II) and organ function as assessed by the Sequential Organ Failure Assessment (SOFA).

### Statistical analysis

Based on previous studies [25,26], a sample size of 279 patients was required to show an increase in the 24-hour success rate from 50% [26] to 70% [25] using prokinetic agent administration, with a two-sided test (α error = 5%; power = 80%). Considering a possible dropout rate of 10%, the trial enrolled 307 patients. All analyses were performed on the per protocol set basis. Demographic data, outcome data and other laboratory parameters are presented as the frequency for categorical variables, and as the mean ± standard deviation or median with interquartile range for continuous variables. Proportions were compared with the chi-square test or Fisher’s exact test, as appropriate. Continuous variables were tested using analysis of variance and Bonferonni *post hoc* test for normally distributed data, or the Wilcoxon rank-sum test for non-normally distributed data. Patients were stratified on a number of baseline covariates such as mechanical ventilation, APACHE II and SOFA scores, use of sedative and analgesic, sex and age. The success rate of post-pyloric placement was compared between different groups with adjustment for baseline covariates. Logistic multivariate stepwise regression (forward) was used to determine the influencing factors. All statistical analyses were performed with SPSS 13.0 (SPSS Inc., Chicago, IL, USA). Two-sided *P* <0.05 was considered statistically significant. *P* <0.016 was considered statistically significant when any two of three groups were compared.

## Results

### Enrollment

**Figure 1:**
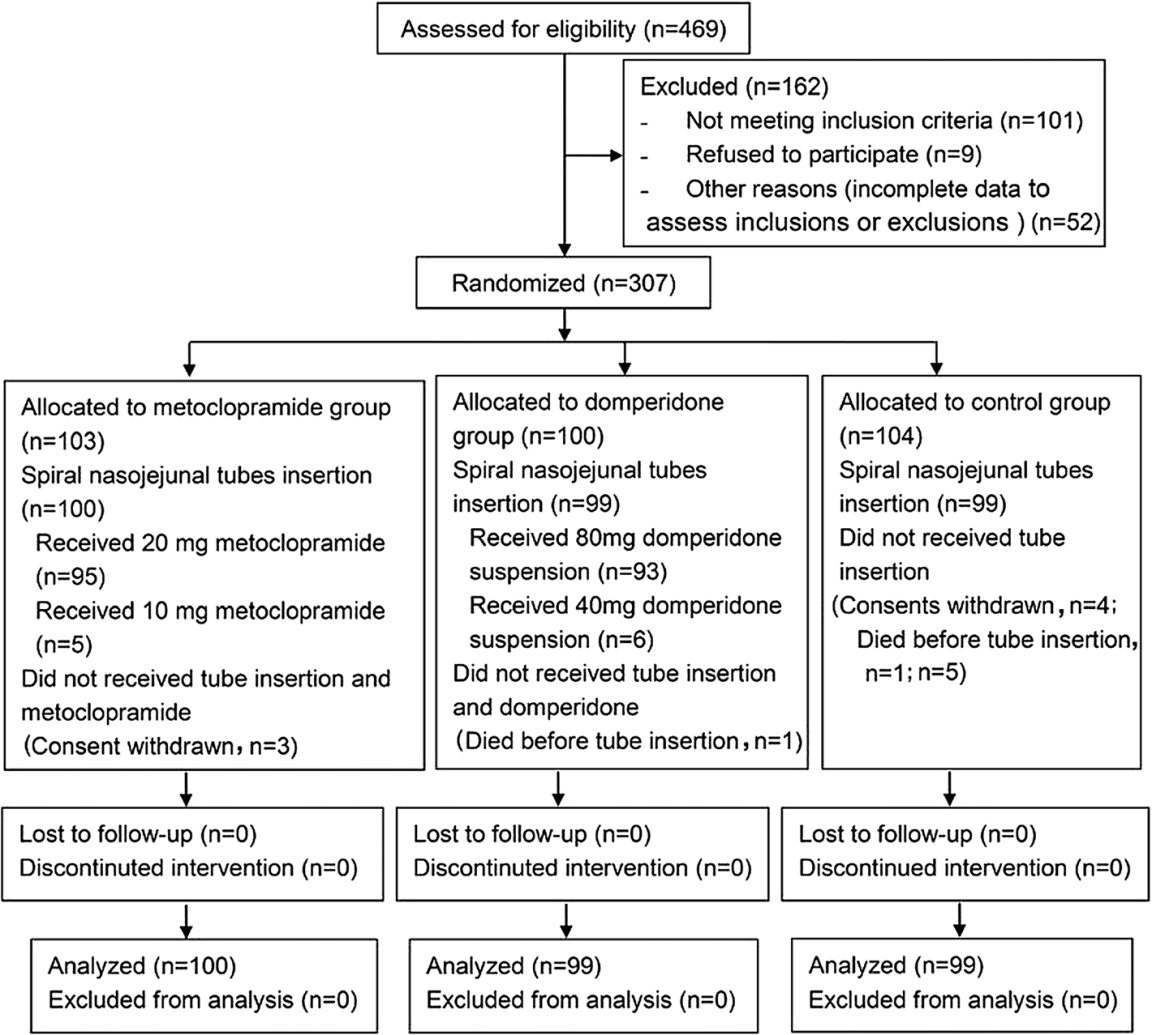
**Study profile** **.**

Between April 2012 and February 2014, 307 eligible patients were randomized (Figure 1). In the metoclopramide group, consent was withdrawn after enrollment in three cases. In the domperidone group, one patient died before tube insertion. In the control group, five patients were excluded: the condition deteriorated rapidly in one patient and he died before insertion; in the other four patients, consent was withdrawn before insertion. Therefore, 298 patients were randomized and underwent tube insertion. Of the 100 patients in the metoclopramide group, 95 patients received 20 mg metoclopramide according to the protocol and five patients with renal insufficiency received 10 mg metoclopramide. In the domperidone group, 93 patients completed the trial in adherence to the protocol, while the remaining six patients received 40 mg domperidone because they were transferred out of the ICU within 24 hours after tube insertion. There was no withdrawal after tube insertion. All patients completed the 24-hour observation and were included in the statistical analyses (Figure 1).

### Baseline data

Most demographic and primary diagnosis characteristics were similar between the three groups (Table 1). Mean age was over 60 years in each group. Respiratory and neurologic diseases were the most common primary diagnoses. Most patients received mechanical ventilation. There was no difference in the frequency of use of sedatives, analgesics, vasopressors and mechanical ventilation. The APACHE II score of each group was >20. APACHE II and SOFA scores were similar between the three groups (*P* >0.05).

**Table 1:** **Patients’ baseline characteristics**

**Variable**	**Metoclopramide group**	**Domperidone group**	**Control group**	***P*** **value**
	**(** ***n*** **= 100)**	**(** ***n*** **= 99)**	**(** ***n*** **= 99)**	
Age (years)				
Average	62.1 ± 18.6	65.2 ± 15.1	61.3 ± 17.4	0.233
Median	64	69	64	
Range	18 to 93	27 to 95	20 to 96	
Male	67 (67.0)	58 (58.6)	72 (72.7)	0.107
Primary diagnosis				
Respiratory	20 (20.0)	18 (18.2)	22 (22.2)	0.777
Cardiovascular	5 (5.0)	10 (10.1)	9 (9.1)	0.375
Neurological	48 (48.0)	41 (41.4)	39 (39.4)	0.439
Sepsis	14 (14.0)	11 (11.1)	13 (13.1)	0.822
Multitrauma	7 (7.0)	6 (6.1)	10 (10.1)	0.537
Other	6 (6.0)	13 (13.1)	6 (6.1)	0.114
Use of sedatives	31 (31.0)	30 (30.3)	29 (29.3)	0.966
Use of analgesics	10 (10.0)	12 (12.1)	9 (9.1)	0.773
Use of vasopressors	24 (24.0)	21 (21.2)	25 (25.3)	0.790
Ventilation	72 (72.0)	66 (66.7)	68 (68.7)	0.713
Average APACHE II score	21.7 ± 7.5	20.7 ± 6.6	21.0 ± 7.3	0.582
< 20	45 (45.0)	43 (43.4)	43 (43.4)	0.967
≥ 20	55 (55.0)	56 (56.6)	56 (56.6)	
Average SOFA score	9.9 ± 2.9	9.7 ± 2.8	9.7 ± 2.9	0.858
< 12	66 (66.0)	68 (68.7)	66 (66.7)	0.916
≥ 12	34 (34.0)	31 (31.3)	33 (33.3)	

Data presented as mean ± standard deviation, interquartile range or *n* (%). APACHE, Acute Physiology and Chronic Health Evaluation; SOFA, Sequential Organ Failure Assessment.

The average time for tube insertion in all patients was 10.3 minutes, with 10.1 minutes in the metoclopramide group, 9.6 minutes in the domperidone group and 11.0 minutes in the control group (*P* >0.05). The spiral nasojejunal tube was successfully inserted into the stomach in 92.2% of all patients after one to three attempts. Only one patient underwent eight attempts before successful placement because of a highly sensitive gag reflex. There was no difference in attempts at tube insertion between the three groups.

### Primary outcome

Twenty-four hours after tube insertion, successful post-pyloric placement was achieved in 55 of 100 patients in the metoclopramide group (55.0%), in 51 of 99 patients in the domperidone group (51.5%) and in 27 of 99 patients in the control group (27.3%) (*P* = 0.0001). The relative probability of success in the metoclopramide group was 3.3 compared with the control group (95% confidence interval (CI): 1.8 to 5.9, *P* = 0.0001). The relative probability of success in the domperidone group was 2.8 compared with the control group (95% CI: 1.6 to 5.1, *P* = 0.0005). There was no difference between the metoclopramide and the domperidone groups.

### Secondary outcomes

The tubes migrated into D1 in eight patients, D2 in 13 patients, D3 in seven patients, D4 in eight patients and proximal jejunum in 19 patients in the metoclopramide group. The proportion of patients with post-D1, post-D2 and post-D3 placement in the metoclopramide group was higher than in the control group. There was no difference in the proportion of patients with tubes migrated into proximal jejunum between the metoclopramide group and the control group (odds ratio (OR) = 2.1; 95% CI: 0.9 to 4.8; *P* = 0.0752). In the domperidone group, the tubes migrated into D1 in seven patients, D2 in four patients, D3 in 14 patients, D4 in 10 patients and proximal jejunum in 16 patients. The proportion of patients with post-D1, post-D2 and post-D3 placement in the domperidone group was higher than in the control group. There was no difference in the proportion of patients with tubes migrated into proximal jejunum between the domperidone and control groups (OR = 1.7; 95% CI: 0.7 to 4.0; *P* = 0.2068) (Table 2).

**Table 2:** **Primary and secondary outcomes**

	**Metoclopramide group**	**Domperidone group**	**Control group**	***P*** **value**
	**(** ***n*** **= 100)**	**(** ***n*** **= 99)**	**(** ***n*** **= 99)**	
Tube tip position				
Post-pyloric	55 (55.0)^**^	51 (51.5)^**^	27 (27.3)	0.0001
Post-D1	47 (47.0)^**^	44 (44.4)^**^	24 (24.2)	0.0015
Post-D2	34 (34.0)^**^	40 (40.4)^**^	13 (13.1)	0.0001
Post-D3	27 (27.0)^*^	26 (26.3)^*^	11 (11.1)	0.0088
Proximal jejunum	19 (19.0)	16 (16.2)	10 (10.1)	0.2016
Tube migration distance				
Initial nose scale (cm)	75.3 ± 7.5	75.1 ± 7.0	75.2 ± 7.1	0.9708
Nose scale at 24 hours (cm)	86.1 ± 10.3^†^	84.9 ± 10.3^†^	80.6 ± 8.9	0.0003
Migration distance (cm)	10.8 ± 8.1^†^	9.8 ± 8.5^†^	5.5 ± 7.6	0.0001

Data presented as mean ± standard deviation or *n* (%). Post-pyloric, reaching the first portion of the duodenum or beyond. Post-D1, reaching the second portion of the duodenum or beyond. Post-D2, reaching the third portion of the duodenum or beyond. Post-D3, reaching the fourth portion of the duodenum or beyond. *P <0.016, metoclopramide group versus controls or domperidone group versus controls. **P <0.003, metoclopramide group versus controls or domperidone group versus controls. ^†^ *P* <0.01, metoclopramide group versus controls or domperidone group versus controls.

There was no significant difference in the initial nose scale of the tubes between the three groups. Twenty-four hours after tube insertion, the average tube migration distance was 10.8 cm (95% CI: 9.2 to 12.4) in the metoclopramide group, 9.8 cm (95% CI: 8.1 to 11.5) in the domperidone group and 5.5 cm (95% CI: 4.0 to 7.0) in the control group (*P* = 0.0001) (Table 2). There was a 5.3 cm (95% CI: 1.9 to 8.7) longer average migration distance in the metoclopramide group compared with the control group (*P* <0.01), and a 4.3 cm longer average migration distance in the domperidone group compared with the control group (*P* <0.01). No significant difference was observed in the migration distance between the metoclopramide and the domperidone groups.

### Safety

Safety of the study drugs and the tubes were assessed in all patients according to all adverse events recorded within 24 hours after tube insertion. There was no difference in the incidence of any adverse events between the three groups (Table 3). The most common drug-related adverse events were lethargy, irritability, muscle tremor and itching. These symptoms disappeared within 24 hours without any treatment. The overall incidence was 1.7%, and there was no difference between the three groups. Nasal mucosa bleeding was the most common tube-related event and was recorded in 21 patients (7.0%). Bleeding stopped spontaneously without any treatment, and there was no difference between the three groups (*P* >0.05). The nasojejunal tube was misplaced into airways in only one patient in the metoclopramide group and one in the control group. The tube was pulled out immediately and did not cause any adverse effect. Other tube-related adverse events included pain, nausea and vomiting, with an overall incidence of 6.0%. There was no difference between the three groups (*P* >0.05).

**Table 3:** **Adverse events**

**Event**	**Metoclopramide group**	**Domperidone group**	**Control group**	***P*** **value**
	**(** ***n*** **= 100)**	**(** ***n*** **= 99)**	**(** ***n*** **= 99)**	
Any event	20 (20.0)	10 (10.1)	13 (13.1)	0.126
Drug-associated events				
Lethargy	2 (2.0)	0	0	0.136
Dysphoria	1 (1.0)	0	0	0.370
Amyostasia	1 (1.0)	0	0	0.370
Pruritus	0	1 (1.0)	0	0.365
Tube-associated events				
Nasal mucosa bleeding	8 (8.0)	5 (5.1)	8 (8.1)	0.637
Airway misplacement	1 (1.0)	0	1 (1.0)	0.606
Pain	2 (2.0)	3 (3.0)	2 (2.0)	0.861
Nausea	4 (4.0)	1 (1.0)	1 (1.0)	0.222
Vomiting	1 (1.0)	1 (1.0)	1 (1.0)	1.000

Data presented as *n* (%).

### Subgroup analyses

Success rates among prespecified subgroups of patients are presented in Table 4. Prespecified analyses of the primary endpoint, where patients were stratified according to APACHE II score, SOFA score, mechanical ventilation, gender and age, showed that both metoclopramide and domperidone tended to improve the success rate of post-pyloric placement. In patients with APACHE II score <20 (*P* <0.016), SOFA score ≥12 (*P* <0.0033), age ≥60 or <60 (both *P* <0.016), non-neurological disease (*P* <0.016), sepsis or not (*P* <0.016), without sedative and analgesic (*P* <0.0033), without vasopressor (*P* <0.0033), without mechanical ventilation (*P* <0.016) and male patients (*P* <0.016), both metoclopramide and domperidone improved the success rate of post-pyloric placement compared with controls. In patients with SOFA score <12 (*P* = 0.036) and neurological disease (*P* = 0.0292), the proportion of post-pyloric placement was higher in the metoclopramide group compared with the domperidone group (Table 4).

**Table 4:** **Analysis of success rate of post-pyloric placement of the nasojejunal tube in prespecified subgroups**

**Subgroup**	**Metoclopramide group (** ***n*** **= 100)**	**Domperidone group (** ***n*** **= 99)**	**Control group (** ***n*** **= 99)**	**Total (** ***n*** **= 298)**	***P*** **value**
Age					
<60 years	22/38 (57.9)^*^	19/35 (54.3)^*^	11/41 (26.8)	52/114 (45.6)	0.0100
≥60 years	33/62 (53.2)^*^	32/64 (50.0)^*^	16/58 (27.6)	81/184 (44.0)	0.0090
Sex					
Male	38/67 (56.7)^**^	28/58 (48.3)^*^	17/72 (23.6)	83/197 (42.1)	0.0002
Female	17/33 (51.5)	23/41 (56.1)	10/27 (37.0)	50/101 (49.5)	0.2944
APACHE II score					
< 20	31/45 (68.9)^**^	27/43 (62.8)^*^	14/43 (32.6)	72/131 (55.0)^#^	0.0013
≥ 20	24/55 (43.6)	24/56 (42.9)	13/56 (23.8)	61/167 (36.5)	0.0398
SOFA score					
< 12	39/66 (59.1)^*^	37/68 (54.4)^‡^	24/66 (36.4)	100/200 (50.0)^†^	0.0221
≥ 12	16/34 (47.1)^**^	14/31 (45.2)^**^	3/33 (9.1)	33/98 (33.7)	0.0012
Neurological diseases					
Yes	27/48 (56.3)^**^	19/41 (46.3)^∫^	9/39 (23.1)	55/128 (43.0)	0.0069
No	28/52 (53.8)^*^	32/58 (55.2)^*^	18/60 (30.0)	78/170 (45.9)	0.0089
Sepsis					
Yes	9/14 (64.3)^*^	7/11 (63.6)^*^	2/13 (15.4)	18/38 (47.4)	0.0173
No	46/86 (53.5)^**^	44/88 (50.0)^*^	25/86 (29.1)	115/260 (44.2)	0.0023
Use of sedatives or analgesics					
Yes	15/35 (42.9)	16/37 (43.2)	12/38 (31.6)	43/110 (39.1)	0.5023
No	40/65 (61.5)^**^	35/62 (56.5)^**^	15/61 (24.6)	90/188 (47.9)	0.0001
Use of vasopressors					
Yes	10/24 (41.7)	8/21 (38.1)	4/25 (16.0)	22/70 (31.4)^¶^	0.1129
No	45/76 (59.2)^**^	43/78 (55.1)^**^	23/74 (31.1)	111/228 (48.7)	0.0009
Mechanical ventilation					
Yes	33/72 (45.8)	31/66 (47.0)	19/68 (27.9)	83/206 (40.3)^‖^	0.0397
No	22/28 (78.6)^**^	20/33 (60.6)^*^	8/31 (25.8)	50/92 (54.3)	0.0002

Data presented as success/total (%). APACHE, Acute Physiology and Chronic Health Evaluation; SOFA, Sequential Organ Failure Assessment. *P <0.016, metoclopramide or domperidone group versus controls. **P <0.0033, metoclopramide or domperidone group versus controls. ^#^ *P* = 0.0015, APACHE II score <20 versus ≥20. ^‡^ *P* = 0.0360, SOFA <12 in the domperidone group versus controls. ^†^ *P* = 0.0077, SOFA score <12 versus ≥12. ^∫^ *P* = 0.0292, neurologic diseases in the domperidone group versus controls. ^¶^ *P* = 0.0111, use of vasopressors versus without vasopressor. ^‖^ *P* = 0.0241, mechanical ventilation versus no ventilation.

### Independent factors influencing insertion success

All patients were divided into two groups according to tube tip position. Patients with tubes in the stomach were assigned to the failure group, and patients with successful post-pyloric tube placement were assigned to the success group. Influencing factors of tube migration were analyzed. The proportion of patients with use of prokinetic agents, APACHE II score <20, SOFA score <12, without vasopressor and without mechanical ventilation in the success group was significantly higher than in the failure group (Table 5). APACHE II and SOFA scores were included in a logistic regression analysis because they were both used to access the severity of the illness. Logistic multiple stepwise regression showed that use of prokinetic agents, APACHE II score <20, SOFA score <12 and without vasopressor were independent influencing factors for the success rate of post-pyloric placement (Table 6).

**Table 5:** **Effects of various factors on the success rate of post-pyloric placement**

**Variable**	**Success group**	**Failure group**	***P*** **value**
	**(** ***n*** **= 133)**	**(** ***n*** **= 165)**	
Age <60 years	52 (39.1)	62 (37.6)	0.7881
Male	83 (62.4)	114 (69.1)	0.2255
Use of prokinetic agents	106 (79.7)	93 (56.4)	0.0001
APACHE II score <20	72 (54.1)	59 (35.8)	0.0015
SOFA score <12	100 (75.2)	100 (60.6)	0.0077
Non-neurological disease	78 (58.6)	92 (55.8)	0.6165
Non-sepsis	115 (86.5)	145 (87.9)	0.7163
Without sedative and analgesic	90 (67.7)	98 (59.4)	0.1411
Without vasopressor	111 (83.5)	117 (70.9)	0.0111
Without mechanical ventilation	50 (37.6)	42 (25.5)	0.0241

Data presented as *n* (%). APACHE, Acute Physiology and Chronic Health Evaluation; SOFA, Sequential Organ Failure Assessment.

**Table 6:** **Multivariate logistic regression analysis of factors for the success of post-pyloric placement**

**Influencing factor**	***P*** **value**	**Odds ratio**	**95% CI**
Including APACHE II score			
Use of prokinetic agents	<0.0001	3.155	1.843-5.401
APACHE II score <20	0.0030	2.098	1.286-3.421
Without vasopressor	0.0323	1.913	1.056-3.465
Including SOFA score			
Use of prokinetic agents	<0.0001	3.116	1.828-5.311
SOFA score <12	0.0223	1.853	1.092-3.146
Without vasopressor	0.0435	1.850	1.018-3.360

Data presented as *n* (%). APACHE, Acute Physiology and Chronic Health Evaluation; CI, confidence interval; SOFA, Sequential Organ Failure Assessment.

## Discussion

This multicenter, randomized, open-label, controlled clinical trial showed that both metoclopramide and domperidone can improve the success rate of post-pyloric placement of spiral nasojejunal tubes in critically ill patients. The success rate in the metoclopramide group (55.0%) and the domperidone group (51.5%) was significantly higher than that in the control group (27.3%). In addition, there was a longer migration distance of the tubes in the metoclopramide and domperidone groups than in the control group. Logistic regression analysis identified use of prokinetic agents, APACHE II score <20, SOFA score <12 and without vasopressor as independent factors influencing the success rate of the feeding tube migration. No serious drug-related adverse reaction was observed.

Metoclopramide and domperidone are commonly used as prokinetic agents in the ICU. Studies have shown that metoclopramide may improve gastrointestinal peristalsis, increase gastric emptying and increase patients’ tolerance to enteral nutrition [27-29], but did not reduce the incidence of ventilation-associated pneumonia [30,31]. Metoclopramide was often used to promote the success rate of bedside blind placement of nasojejunal tubes [32,33], but its effects are controversial. An early prospective controlled study showed that intravenous 10 mg metoclopramide administered 10 minutes prior to intubation with a small-bore feeding tube was ineffective in facilitating transpyloric intubation [23]. Another study demonstrated that metoclopramide administered after nasogastric intubation was ineffective in promoting transpyloric advancement of feeding tubes, but that there was a significant increase in transpyloric intubation when metoclopramide was administered prior to tube insertion [33]. A small clinical trial by Lai and colleagues showed that 10 mg metoclopramide administered prior to intubation achieved a success rate of 57% in patients with gastric motility disorders [18]. A review including four studies demonstrated that there was no statistically significant difference between intravenous or intramuscular metoclopramide administered to promote tube migration (OR = 0.65, 95% CI: 0.33 to 1.28), and that intravenous 10 mg metoclopramide (OR = 0.68, 95% CI: 0.37 to 1.23) and 20 mg metoclopramide (OR = 0.27, 95% CI: 0.01 to 10.84) were equally ineffective in facilitating transpyloric intubation [34]. The present large trial showed that the success rate of post-pyloric placement of nasojejunal tube increased significantly with administration of metoclopramide. This is evidence supporting the use of metoclopramide in spiral nasojejunal tube insertion.

A number of clinical trials showed that domperidone significantly improves patients’ gastrointestinal motility, especially in diabetic patients with gastric paralysis. Although domperidone is approved by the Chinese Drugs Agency, it is not approved by the US Food and Drug Administration to be used widely because of its potential cardiac toxicity. A systemic evaluation by Sugumar and colleagues suggested that domperidone may improve the symptoms of diabetic patients with gastric paralysis by improving gastric emptying [35]. However, there has been no relevant study evaluating whether domperidone can improve gastrointestinal motility and its effects on the success rate of nasojejunal tube placement in critically ill patients. In the present trial, the success rate of post-pyloric placement of a spiral nasojejunal tube in the domperidone group was 51.5%, similar to that in the metoclopramide group (55.0%) and significantly higher than in the control group (27.3%). Moreover, the proportion of post-D1, post-D2 and post-D3 placement was also higher than in the control group. The tubes migrated for longer distances than in the control group in both the metoclopramide and domperidone groups. These results strongly suggest that domperidone is also a candidate as a prokinetic agent for post-pyloric placement of a spiral nasojejunal tube in critical illness.

In the present study, there was also no difference in the success rate of post-D1, post-D2, post-D3 and proximal jejunum placement between the metoclopramide group and the domperidone group. These results indicate that the effect of domperidone may be similar to metoclopramide in improving the success rate of spiral nasojejunal tube insertion. However, the sample size of this study was not enough to detect possible differences between the two agents in improving post-pyloric migration placement. A non-inferiority trial would be needed.

Erythromycin is another prokinetic agent commonly used in the clinical setting and is often used to promote the migration of spiral nasojejunal tubes. Early studies suggested that erythromycin was effective in facilitating the bedside placement of nasoenteric feeding tubes into the duodenum in ICU patients [36,37]. However, some studies observed different results. A randomized, double-blind, placebo-controlled study in critical pediatric patients by Gharpure and colleagues showed that erythromycin did not facilitate the transpyloric passage of feeding tubes in critically ill children. In addition, the distal migration of duodenal tubes further into the small bowel was not enhanced by erythromycin [38]. A randomized, controlled trial including 40 subjects failed to determine any benefit of erythromycin in terms of success or time of migration to jejunal position using a self-propelled feeding tube [26]. Another randomized study showed that erythromycin was more effective than metoclopramide in treating feed intolerance, but that there was a rapid decline in both treatments with time [39]. The reasons for these differences may include patients’ heterogeneity, different tubes used and tolerance to drugs [26], and also the fact that we observed only the short-term efficacy for tube placement. In addition, clinicians have to consider whether low-dose erythromycin could induce bacterial drug resistance. Erythromycin was therefore not selected as a prokinetic agent in the present trial.

A retrospective analysis of 428 patients performed by Metheny and colleagues found that a feeding tube in the mid-duodenum and beyond could reduce the risk of aspiration and associated pneumonia [40]. In the present trial, the proportion of feeding tubes in the post-D1, post-D2 and post-D3 position was 47.0%, 34.0% and 27.0%, respectively, in the metoclopramide group, all significantly higher than in the control group. The proportion of feeding tubes in the post-D1, post-D2 and post-D3 position was 44.4%, 40.4% and 26.3%, respectively, in the domperidone group, all significantly higher than in the control group. These results indicated that the use of either metoclopramide or domperidone for assistance of spiral nasojejunal tube insertion may reduce the risk of aspiration and the incidence of hospital-acquired pneumonia. It must be noted that the low proportion of nasojejunal tube migration into the jejunum in the present trial could indicate that spiral nasojejunal tubes may not be the preferred route for patients with diseases requiring strict jejunum feeding, such as acute pancreatitis.

Karsenti and colleagues found that the ligament of Treitz was reached in a median of 12 hours (range 1 to 96 hours) when the nasoenteric Flocare tube was used for severe acute pancreatitis [41]. Berger and colleagues found that the success rate of post-pyloric placement was 40% at 24 hours after insertion, while a success rate of 58% was achieved at 72 hours [17]. However, a study by van den Bosch and colleagues indicated that the nasojejunal tube did not migrate beyond the pylorus although the observation time was extended to 48 hours in patients with first failed insertion [26]. In addition, nutrition support guidelines by the Society of Critical Care Medicine and the American Society for Parenteral and Enteral Nutrition in critically ill adult patients with intolerance to gastric feeding suggest that enteral nutrition should be initiated within 24 to 48 hours after admission to the ICU, and that the nutrition goal should be achieved within 48 to 72 hours [42]. Therefore, in the present trial, tube tip positions were confirmed by abdominal X-ray scan 24 hours after insertion in all patients.

Complications associated with nasojejunal tubes include inadvertent misplacement of the tube, epistaxis, sinusitis, inadvertent tube removal, tube clogging, tube-feeding-associated diarrhea and aspiration pneumonia [43]. The incidence of airway misplacement of feeding tubes at a major tertiary referral university hospital was 3.2% [44]. Duodenal perforation due to a kink in a nasojejunal feeding tube in a patient with severe acute pancreatitis was also reported [45]. Nasal mucosa bleeding was the most common adverse event associated with tube insertion, with an incidence of 7.0%, in the present study. The bleeding stopped spontaneously without any treatment. The tubes were misplaced into airways in two patients and removed immediately. No serious adverse effect on the patients was observed due to the misplacement. A slight muscle tremor was observed in only one patient after administration of metoclopramide. The symptom disappeared without any treatment. No cardiac adverse effect was observed in all patients who received domperidone. The low incidence (1.7%) of suspected drug-related adverse events, without significant difference from the control group, indicated that metoclopramide and domperidone were safe using the doses used in the present study. However, subjective sensations such as headache, severe thirst, and pronunciation difficulties were difficult to assess due to the severity of disease, sedation or analgesia and mechanical ventilation.

The effects of metoclopramide and domperidone on the success rate of post-pyloric placement of spiral nasojejunal tubes were analyzed in all subgroups including age, sex, APACHE II score, SOFA score, diagnosis of neurologic diseases and sepsis, and use of sedatives, analgesics and vasopressors. The aim of analyzing different prespecified subgroups was in preparation for a future study targeting specific groups of critically ill patients who might benefit from prokinetic agents, because it is unlikely that prokinetic agents are equally beneficial to all patients in view of the significant heterogeneity in patients’ characteristics, severity of illness and treatments in critically ill patients. Analysis of the current study showed that patients with APACHE II score <20, SOFA score ≥12, age ≥60 or <60, non-neurological disease, sepsis or not, without mechanical ventilation, without sedative and analgesic, without vasopressor and male patients benefited from the use of prokinetic agents, which provides evidence for tailored therapy. The results of subgroup analyses in the present study were inconclusive and whether prokinetic agents are more effective in specific groups of patients with spiral nasojejunal tube placement in the ICU should be explored in trials with larger sample sizes.

All patients were divided according to success or failure of tube tip migration beyond the pylorus. Logistic regression analysis found that use of prokinetic agents, APACHE II score <20, SOFA score <12 and without vasopressor were independent factors influencing the success rate of tube insertion, which was consistent with a study by Berger and colleagues [17]. The success rate of post-pyloric placement of a spiral nasojejunal tube may be relatively high in patients with the above factors. The higher success of this study in patients with lower disease severity may suggest that this drug/tube technique may be less successful in those with greatest need for intestinal feeding. Therefore, spiral nasojejunal tubes may be the preferred and most reliable route for enteral nutrition in such patients.

A recent study has suggested that electromagnetic guiding of the tube resulted in a high placement success rate (97.2% vs. 27 to 55% in the present study) [46]. This could replace the X-ray confirmation of tube placement since about 10.5% of the radiologic interpretations can be inaccurate [47]. The use of the electromagnetic guiding resulted in success rates that were comparable with endoscopic placement [48]. Further studies will be required to compare these technologies with the use of prokinetic agents. However, limited access to new technologies might be an impediment to their implementation in some countries.

To the best of our knowledge, this is the first multicenter, randomized, controlled trial to confirm whether prokinetic agents can promote the success rate of post-pyloric placement of self-propelling spiral nasojejunal feeding tubes. To minimize the potential bias, randomization was conducted in the study, but fixed block randomization was used, which could introduce a bias if the block size is guessed. Meanwhile, the radiograph of each patient was assessed by an expert group of ICU clinicians and radiologists who were independent and blinded to the treatment allocation, thus reducing the bias to a great extent and increasing the validity of the results. The reason for the lower success rate of post-pyloric placement of nasojejunal tubes compared with some previous studies may be explained by the severity of patients enrolled in this trial. In the present study, the absence of double-blinding limits the extent to which the results can be generalized. Double-blinding could not be used because of the absence of a placebo with an identical appearance to domperidone suspension, and because of the different dosing regimen of the two drugs. Only the patients and the statistician were blinded. In addition, we only examined one type of tube (Flocare Bengmark; Nutricia), while a previous study showed that other types of tubes (such as the Tiger tube; Cook, Bjaeverskov, Denmark) might result in a higher efficacy [49]. Moreover, all patients who underwent major surgery were excluded, limiting the number of patients despite the large number of participating hospitals. Finally, we included all patients, irrespective of their gastric emptying status. Further studies should be conducted in patients stratified based on gastric emptying.

## Conclusions

The present trial strongly suggests that either metoclopramide or domperidone may facilitate post-pyloric placement of spiral nasojejunal tubes in critically ill patients. Either metoclopramide or domperidone may facilitate post-D1, post-D2 and post-D3 placement of spiral nasojejunal tubes. No serious adverse event was observed with administration of metoclopramide or domperidone. Use of prokinetic agents, APACHE II score <20, SOFA score <12 and without vasopressor were independent factors influencing the success rate of tube insertion.

## Key messages

- Either metoclopramide or domperidone may facilitate post-pyloric placement of spiral nasojejunal tubes in critically ill patients.
- Either metoclopramide or domperidone may facilitate post-D1, post-D2 and post-D3 placement of spiral nasojejunal tubes.
- No serious adverse event was observed with administration of metoclopramide or domperidone.

## Abbreviations

APACHE: Acute Physiology and Chronic Health Evaluation
CI: confidence interval
D1: first portion of the duodenum
D2: second portion of the duodenum
D3: third portion of the duodenum
D4: fourth portion of the duodenum
OR: odds ratio
SOFA: Sequential Organ Failure Assessment
